# Highly Sensitive and Selective Eco-Toxic 4-Nitrophenol Chemical Sensor Based on Ag-Doped ZnO Nanoflowers Decorated with Nanosheets

**DOI:** 10.3390/molecules26154619

**Published:** 2021-07-30

**Authors:** Ahmad Umar, M. Shaheer Akhtar, Hassan Algadi, Ahmed A. Ibrahim, Mohsen A. M. Alhamami, Sotirios Baskoutas

**Affiliations:** 1 Department of Chemistry, Faculty of Science and Arts, Najran University, P.O. Box 1988, Najran 11001, Saudi Arabia; ahmedragal@yahoo.com (A.A.I.); maalhammami@nu.edu.sa (M.A.M.A.); 2 Promising Centre for Sensors and Electronic Devices (PCSED), Najran University, P.O. Box 1988, Najran 11001, Saudi Arabia; hassan.algadi@gmail.com; 3 New & Renewable Energy Material Development Center (NewREC), Jeonbuk National University, Jeonju-si, Jeollabuk-do 54896, Korea; shaheerakhtar@jbnu.ac.kr; 4 Department of Electrical Engineering, Faculty of Engineering, Najran University, P.O. Box 1988, Najran 11001, Saudi Arabia; 5 Department of Materials Science, University of Patras, GR-26504 Patras, Greece

**Keywords:** Ag-doped ZnO, nanoflowers, electrochemical sensor, 4-nitrophenol, oxidation

## Abstract

Herein, we have developed a novel sensing electrode to detect the eco-toxic 4-nitrophenol (4-NP). Ag-doped-ZnO nanoflowers were synthesized by facile hydrothermal method and examined by several characterization techniques in order to understand the morphology, crystal structure, composition, and surface properties. Morphological results were confirmed by the formation of Ag-doped ZnO nanoflowers decorated with nanosheets. Ag-doped ZnO/glassy carbon electrode (GCE) electrode-material-matrix was used for electrochemical sensing of toxic 4-NP. Under optimized conditions, Ag-doped ZnO/GCE modified electrode exhibits high-sensitivity and selectivity compared to the bare GCE electrode. The Ag-doped ZnO/GCE modified electrode exhibits high electrocatalytic oxidation towards 4-NP. Anodic peak current of 4-NP is increased linearly by increasing the concentration of nitrophenol. Additionally, Ag-doped ZnO/GCE shows a wide range of sensitivity from 10 µM to 500 µM, and a linear calibration plot with a good detection limit of 3 µM (S/N = 3). The proposed Ag-doped ZnO/GCE modified electrode showed high sensing stability. In addition, the oxidation mechanism was studied. The obtained results revealed that the Ag-ZnO/GCE electrode could be the promising sensing electrode for 4-NP sensing.

## 1. Introduction

Detecting nitro aromatic compounds is extraordinarily significant in assessing the danger of various natural tests such as wastewater from mining, paint and drug enterprises, petrochemical items, or the assembling of pesticides [1,2,3,4,5], which have a tendency to damage the living organism and plants in low concentrations [6]. Mainly, nitrophenol is formed during the hydrolysis process of diethyl parathion, which is reported by the United State Environmental Protection Agency (USEPA). Nitrophenol is a common intermediate product used for the production of analgesic, dyes, medicines, leather products, and so on. It is considered a hazardous pollutant in food chains such as fruits, and cultivation crops [7,8,9,10]. Nitrophenol compounds also cause severe environmental pollution, owing to their highly toxic level for humans and animals, and thus have been included in the list of toxic and harmful pollutants in different countries. Because of this, more sensitive and accurate detection of nitrophenol is important and crucial [11].

Several different methods were used for efficient detection of nitro compounds, to name a few, fluorescence, capillary zone electrophoresis, spectrophotometry, HPLC, electrochemical analysis, and so on [12,13,14,15,16]. Among all the methods, the electrochemical analysis is given more consideration due to its outstanding sensitivity, selectivity, cost effective and easy operation techniques [17]. Still, several reports demonstrated oxidation and reduction of nitrophenol with different electrode materials such as carbon materials (carbon nanotubes, graphene, carbon dots, etc.), mesoporous and microporous carbon graphene, metal and metal oxide nanomaterials and their composites, metal sulphides and so on [18,19,20,21]. Nevertheless, the majority of researchers focused on the development of electrode materials for better electrocatalytic activity for nitrophenol detection with high selectivity and sensitivity. However, the metallic nanoparticles had outstanding electrical conductivity and catalytic activity towards chemical processes. Gold nanoparticles have a smaller particle size, better electrical conductivity and outstanding surface roughness. However, the scarcity and cost of gold nanoparticles limits the practical applications. Hence, the development of a novel and low-cost sensing material is highly desirable [22].

In this paper, we report the synthesis of Ag-doped ZnO nanoflowers decorated with nanosheets and their sensing ability of toxic 4-nitrophenol. The synthesized material was examined using several techniques in order to understand the morphology, crystal structure, composition, and surface properties. Ag-doped ZnO nanoflowers decorated with nanosheets were used as an electrode-material-matrix to fabricate electrochemical sensors which exhibited high-sensitivity and selectivity towards 4-nitrophenol. Under optimized conditions, the Ag-doped ZnO/GCE modified electrode exhibits high-sensitivity and selectivity compared to the bare GCE electrode. Additionally, Ag-doped ZnO/GCE showed a wide range of sensitivity from 10 µM to 500 µM and a linear calibration plot with a good detection limit of 3 µM (S/N = 3). In addition, the oxidation mechanism was studied. The obtained results revealed that the Ag-ZnO/GCE electrode could be the promising sensing electrode for 4-NP sensing.

## 2. Results and Discussion

### 2.1. Characterizations and Properties of Ag-Doped ZnO Nanoflowers Decorated with Nanosheets

**Figure 1:**
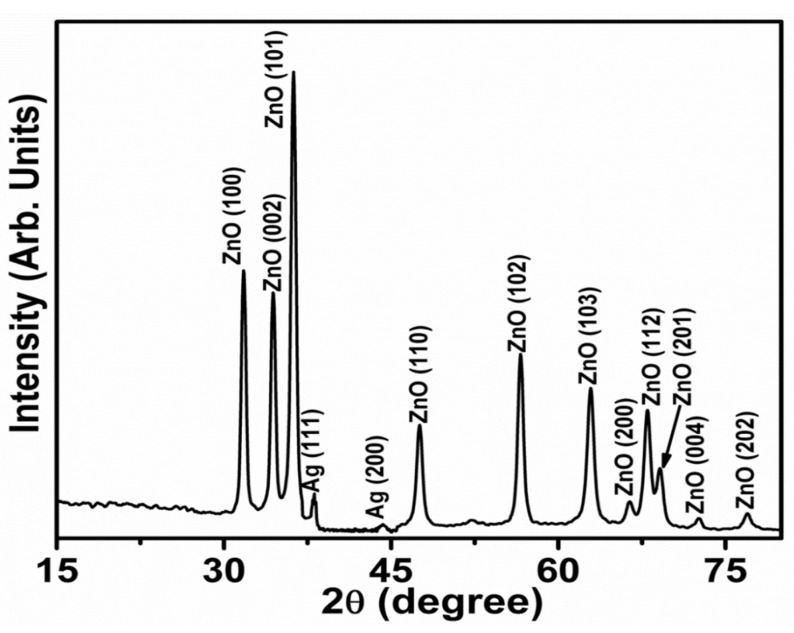
Powder X-ray diffraction pattern of Ag-doped ZnO nanoflowers.

Crystal structure, planes, and phases of the produced Ag-doped ZnO nanoflowers decorated with nanosheets were determined using the X-ray diffraction technique. Figure 1 exhibits the typical XRD pattern of as-synthesized Ag-doped ZnO nanoflowers decorated with nanosheets. Several well-defined diffraction peaks were identified in the observed diffraction pattern, which were closely associated with the wurtzite hexagonal phase of ZnO. The diffraction peaks seen at 2θ = 31.7°, 34.3°, 36.3°, 47.5°, 56.4°, 62.9°, 66.5°, 68.0°, 69.1°, 72.6° and 77.1° can be assigned to various diffraction planes of ZnO (100), (002), (101), (102), (110), (103), (200), (112), (201), (004) and (202), respectively. All the observed diffraction peaks were consistent with the JCPDS card no. 36-1451 and reported literature related to the wurtzite hexagonal phase of ZnO [23,24,25]. Furthermore, two other diffraction peaks appeared at 2θ = 38.1° and 44.3°which can be assigned to diffraction planes of Ag (111) and (200), respectively, and exhibit the Ag face-centered-cubic (fcc) crystal structure. The observed Ag peaks are consistent with the reported literature and JCPDS data card no. 04-0783 [26,27].

**Figure 2:**
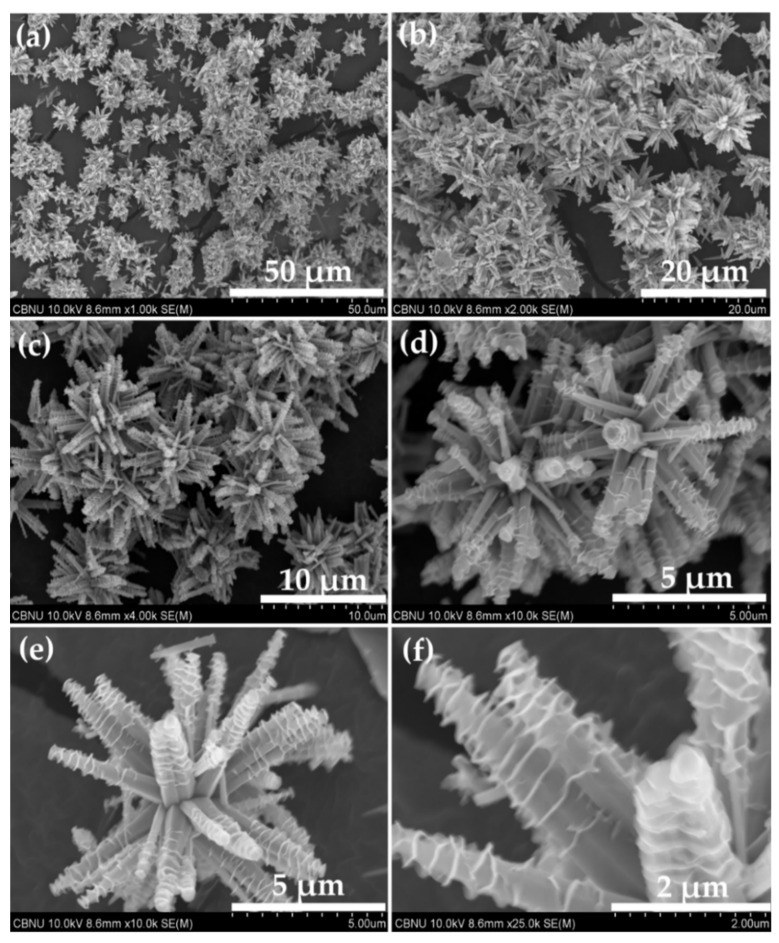
FESEM micrographs of synthesized Ag-doped ZnO nanoflowers decorated with nanosheets.

Surface morphology of the Ag-doped ZnO nanomaterial was scrutinized by FESEM. The findings are displayed in Figure 2. The synthesized material had flower-shaped morphologies, grown in very high density, as evidenced by low-magnification FESEM images (Figure 2a–c). The flower-shaped morphologies were composed of well-defined nanorods which seem to originate from a single nucleus (Figure 2d–f). The hexagonal nanorods possessed wider bases and narrower tips. The typical lengths and diameters of the nanorods were in the range of 6 ± 1 µm and 150 ± 30 nm, respectively.

Interestingly, it was observed that the hexagonal nanorods were decorated with sheet-like morphologies (Figure 2e,f). The thickness of the nanosheets was around 10–15 nm. The nanosheets were precisely wrapped on the nanorods’ surfaces. The typical size of a single flower-shaped structure was 10 ± 2 µm.

**Figure 3:**
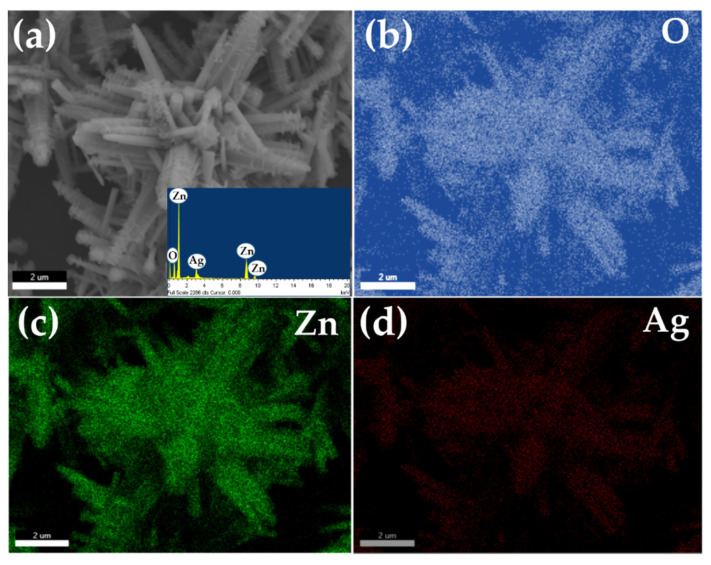
Typical (**a**) SEM image (inset: EDS spectrum) (**b**–**d**) O, Zn and Ag elemental mapping of synthesized Ag-doped ZnO nanoflowers.

The elemental composition of the synthesized Ag-doped ZnO nanoflowers decorated with nanosheets was examined by energy dispersive spectroscopy and elemental mapping. Figure 3a depicts the typical SEM and (b–d) their corresponding elemental mapping images. The EDS spectrum of synthesized Ag-doped ZnO nanoflowers is shown in the inset of Figure 3a, confirming that the material is only composed of zinc (Zn), oxygen (O), and silver. There were no other peaks in the EDS spectrum were related to any other element, confirming that the material is made of Zn, O, and Ag only, without other elements. Figure 3b–d exhibits the elemental mapping of Ag-doped ZnO flower shaped structures. It is clear from the observed elemental mapping that the number of spots corresponding to O and Zn are homogeneous and higher in number over the whole structure, confirmed by the uniform distribution of Zn and O over the whole crystals of flower-shaped structures (Figure 3b,c). The low number of spots in the elemental scan of Ag in the corresponding flower-shaped morphology revealed that Ag is well doped into the lattices of the synthesized material (Figure 3d). Hence, the observed elemental mapping results proved the formation of Ag-doped ZnO.

**Figure 4:**
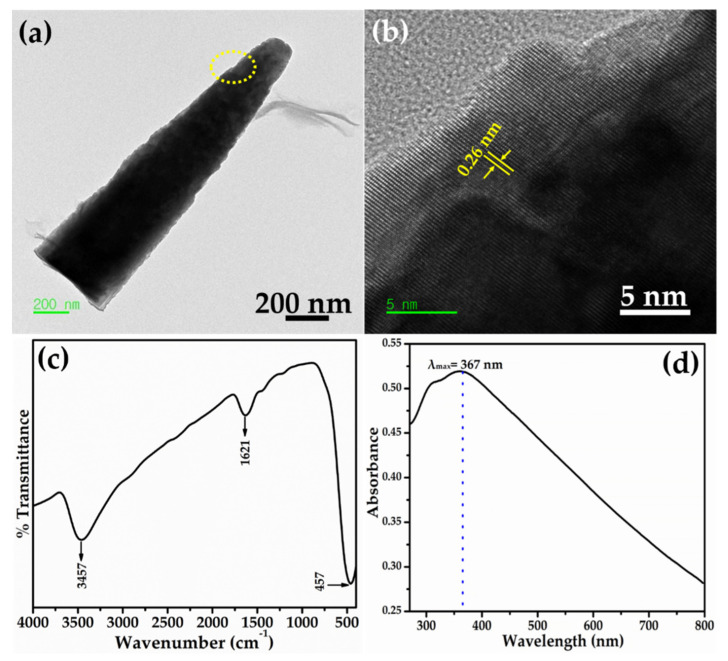
(**a**) HR-TEM image, (**b**) lattice fringes, (**c**,**d**) FTIR and UV-visible spectra of Ag-doped ZnO nanoflowers decorated with nanosheets.

Further detailed morphology and structural features of synthesized Ag-doped ZnO nanoflowers decorated with nanosheets were monitored by HR-TEM. The synthesized material was dispersed in acetone and ultrasonicated for 5 min for TEM examination. Consequently, a drop of acetone containing the synthesized material was placed on the TEM grid and examined. The flower-shaped morphology was torn down into nanorods with ultrasonication. Figure 4a depicts the typical TEM image of a single nanorod. The morphology of the nanorod is consistent with the observed FESEM results, i.e., a sharpened tip with a wider base. Interestingly, the nanorod showed the decoration of nanosheets over its surface. The HR-TEM image, obtained from the circled portion of a single nanorod shown in Figure 4a, clearly displayed well-defined lattice fringes with a d-spacing value between two fringes of 0.26 nm, corresponding to the interplanar spacing of the (0002) plane of wurtzite hexagonal phase ZnO (Figure 4b) [28]. The well-defined lattice fringes confirmed the formation of well-crystalline Ag-doped ZnO nanoflowers.

The metal bonding and functional groups of the Ag-doped ZnO nanoflowers decorated with nanosheets was measured using Fourier transform infrared (FTIR) spectroscopy. Figure 4c demonstrates the typical FTIR spectrum of synthesized Ag-doped ZnO nanoflowers. Few well-defined peaks are observed in the FTIR spectrum at 457 cm^−1^, 1621 cm^−1^, and 3457 cm^−1^. The band at 457 cm^−1^ corresponded to the metal–oxygen (M-O) stretching vibration, confirming the formation of Ag-ZnO nanomaterial [28]. The water O-H stretching and bending frequency was observed at a higher frequency which, due to absorbed water molecules on the surface of the Ag-doped ZnO nanoflowers, may be responsible for the well-defined IR bands at 1621 cm^−1^ and 3457 cm^−1^ [29].

Figure 4d shows the UV-visible spectrum of synthesized Ag-doped ZnO nanoflowers. An intense absorption peak was observed at 367 nm. The band gap energy was calculated according to Equation (1):

(1) $$E_{g}=\frac{1240}{\lambda \max}$$

According to Equation (1), the calculated band gap energy of the synthesized Ag-doped ZnO nanoflowers was 3.38 eV, within the ideal band gap of wurtzite hexagonal phase ZnO [30,31].

**Figure 5:**
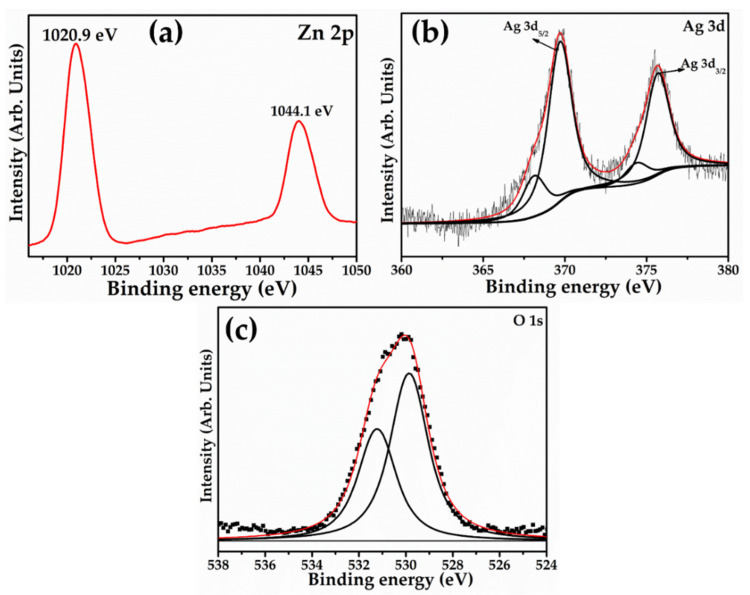
Typical XPS spectra of (**a**) Zn 2p, (**b**) Ag 3d, and (**c**) O 1s of synthesized Ag-doped ZnO nanoflowers decorated with nanosheets.

The chemical compositions and oxidation states of Ag-doped ZnO nanoflowers were studied using X-ray photoelectron spectroscopy (XPS), as shown in Figure 5. To avoid surface charging, all binding energies were corrected using the referenced neutral carbon peak C 1s at 284.8 eV. Doublet binding energies appeared at 1020.9 and 1044.1 eV, which connect to Zn 2p_3/2_ and Zn 2p_1/2_ spin splitting, respectively, in the high-resolution Zn 2p XPS spectrum (Figure 5a). The spin splitting difference of the Zn 2p_1/2_ and Zn 2p_3/2_ peaks was estimated to be 23.2 eV for Ag-doped ZnO nanoflowers, which is close to Zn^2+^ in ZnO [32]. Figure 5b depicts the resolved Ag 3d XPS spectrum, which demonstrates that doublet binding energies appeared at 374.9 and 368.7 eV, corresponding to the Ag 3d_3/2_ and Ag 3d_5/2_ spin splitting, respectively. The presence of Ag 3d_5/2_ with a binding energy of 368.7 eV is related to the metallic Ag; however, it is closer to the binding energy of Ag+ (i.e., 367.9 eV), clearly indicating the presence of Ag+ in ZnO [33]. Because of the valence state difference between Ag^+^ and Zn^2+^ ions, the replacement of Ag^+^ at Zn^2+^ sites in ZnO crystals tends to form zinc interstitial (Zn_i_) or Vo. This caused a reduction in the real doping amount of Ag^+^, which reduced the Zn_i_ or Vo contents. The resolved O 1s XPS spectrum was analyzed in order to understand the oxide bonding in the synthesized material. The observed O1s spectrum exhibited two fitted XPS peaks with binding energies of 529.2 and 531.8 eV (Figure 5c). The peak at 529.2 eV could be related to the O^2−^ ions in Zn-O, revealing the formation of ZnO in Ag-doped ZnO, while the peak at 531.8 eV can be attributed to the adsorbed moisture over the materials surface, which might generate structural defects [34,35].

### 2.2. Electrochemical Activity of Ag-ZnO/GCE Electrode

**Figure 6:**
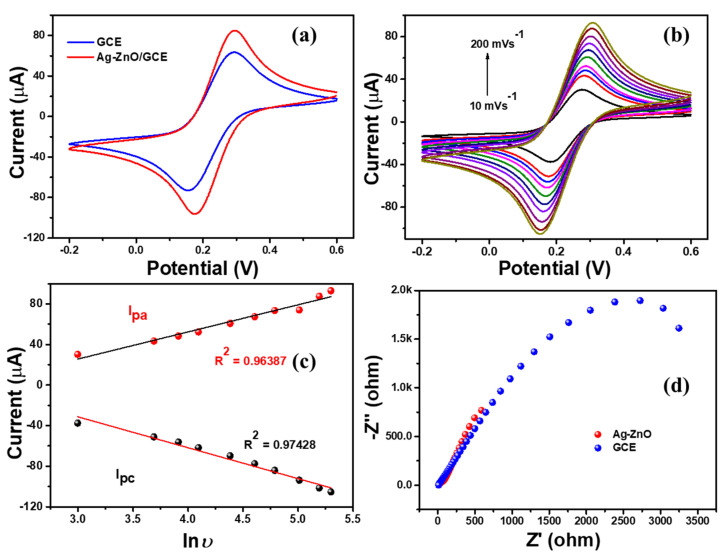
(**a**) CV responses of bare GCE and Ag-ZnO/GCE, (**b**) scan rate variation of Ag-ZnO/GCE, (**c**) linear regression plot (**d**) Nyquist plot for bare GCE and Ag-ZnO/GCE electrode with 5 mM K_3_ [Fe(CN)_6_] in 0.1 M KCl electrolyte.

Electrochemical activity of the Ag-ZnO/GCE modified electrode was analyzed using cyclic voltammetry technique. Briefly, Ag-ZnO modified glassy carbon electrode (Ag-ZnO/GCE) was tested with 5 mM of ferri–ferro redox couple in 0.1 M KCl solution. Figure 6a demonstrated the standard ferri–ferro redox couple of GCE and Ag-ZnO/GCE electrodes. The bare GCE electrode showed the defined redox peaks with the 60 µA peak current, whereas the Ag-ZnO/GCE modified electrode showed a well-defined reversible redox couple with high anodic and cathodic peak current. This was due to the high electron transfer kinetics between the electrode and electrolyte. Moreover, the Ag doping enhanced the electron transfer rate, and the effect of rate variation (10 mVs^−1^ to 200 mVs^−1^) on the electrical activity of the Ag-ZnO/GCE electrode in ferri-ferro solution (Figure 6b). The obtained results revealed that redox peak current linearly increased by increasing the scan rate as shown in Figure 6c. Furthermore, electrochemical impedance spectroscopy (EIS) was used to monitor the resistance of the Ag-ZnO-GCE electrode in ferri-ferro solution.

Figure 6d shows the Nyquist plot of the GCE and Ag-ZnO/GCE electrode. The bare GCE electrode showed a higher resistance R_ct_ value of 2 k ohm; after the modification with Ag-ZnO nanoflowers, it showed a lower resistance value of 0.5 k ohm, which confirms the higher electron transfer capacity of the Ag-ZnO/GCE electrode. Hence, the EIS results revealed that Ag-ZnO/GCE is the convenient electrode for electrochemical sensing applications.

### 2.3. Sensing Applications of Ag-Doped ZnO Nanoflowers

**Figure 7:**
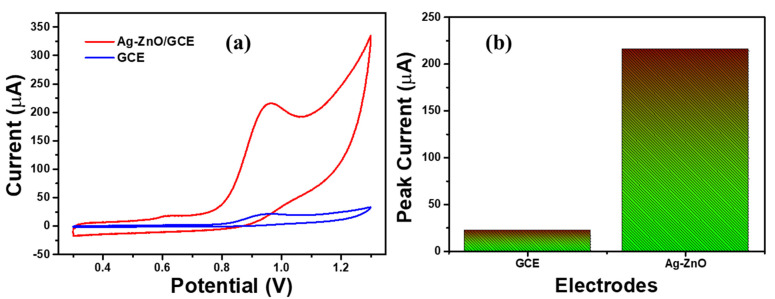
(**a**) CV responses of bare GCE and Ag-ZnO/GCE (**b**) 4-NP sensing activity of the bare GCE and Ag-ZnO/GCE electrode with presence of 500 µM 4-NP containing 0.1 M PBS (pH = 7) solution. (Accumulation time: 2 min, catalyst loading volume: 10 µL).

The electrochemical sensing activity of bare GCE and Ag-ZnO/GCE electrode was investigated with 0.5 µM 4-NP containing 0.1 M PBS solution at a scan rate of 100 mVs^−1^. Figure 7a shows the CV responses of bare GCE and Ag-ZnO/GCE with 500 µM 4-NP containing 0.1 M PBS solution. Bare GCE showed the weak oxidation peak of 4-NP at 0.9 V with poor peak current, which confirms the poor catalytic activity of the bare GCE electrode [36,37].

**Figure 8:**
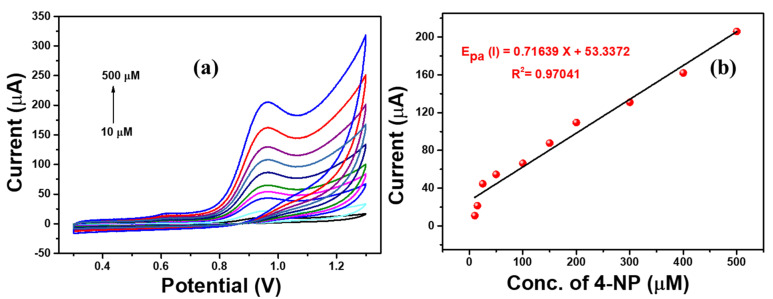
(**a**) CV responses of Ag-ZnO/GCE at different 4-NP concentrations (10 µM to 500 µM) containing 0.1 M PBS (pH = 7) solution at a scan rate of 100 mVs^−1^. (**b**) Linear regression plot of current vs. 4-NP concentration. (Accumulation time: 2 min, catalyst loading volume: 10 µL).

The Ag-ZnO/GCE electrode showed well defined oxidation peak of 4-NP with huge peak current. This confirms the excellent catalytic activity toward 4-NP sensing. In addition, the 4-NP peak current of both electrodes was plotted, and is shown in Figure 7b. This showed excellent sensing activity due to the synergistic of the Ag-doped ZnO nanoflowers. Furthermore, sensitivity of the Ag-ZnO/GCE electrode was analyzed with different concentrations ranging from 10 µM to 500 µM 4-NP solution in 0.1 M PBS solution as shown in Figure 8. The oxidation peak current linearly increased with respect to the concentration of 4-NP. In addition, the calibration plot was fitted against the peak current vs. concentration of 4-NP. This confirmed the sensitivity of Ag-ZnO/GCE towards 4-NP in neutral medium.

**Figure 9:**
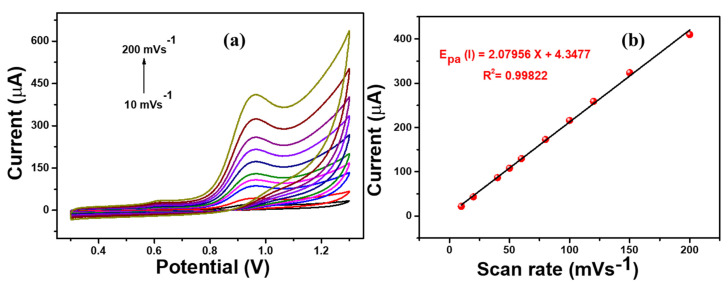
(**a**) CV responses of Ag-ZnO/GCE at different scan rate variations (10 mVs^−1^ to 200 mVs^−1^), (**b**) linear regression plot of current vs. scan rate in presence of 500 µM 4-NP containing 0.1 M PBS (pH = 7) solution. (Accumulation time: 2 min, catalyst loading volume: 10 µL).

In addition, the effect of the scan rate on the 4-NP sensing of Ag-ZnO/GCE was analyzed by varying the applied scan rate from 10 mVs^−1^ to 200 mVs^−1^ with the presence of 500 µM 4-NP containing 0.1 M PBS (pH = 7) solution. Figure 9 shows that the oxidation peak current increased linearly with increasing scan rate. In addition, the peak current was plotted against the scan, is shown in Figure 9b, which suggested that the 4- NP oxidation is a diffusion-controlled process [38,39].

### 2.4. Linear Sweep Voltammetry (LSV) Analysis of 4-NP

**Figure 10:**
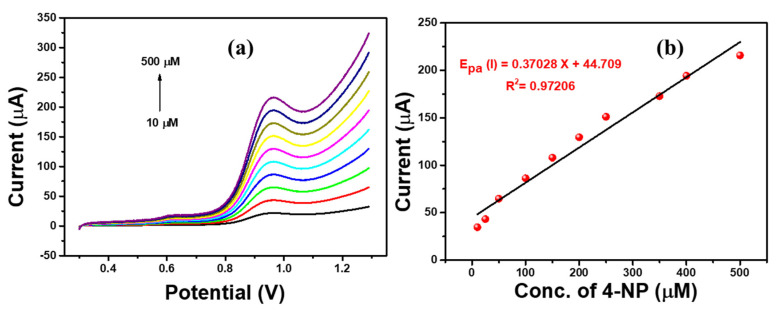
(**a**) LSV responses of Ag-ZnO/GCE at different 4-NP concentrations (10 µM to 500 µM) containing 0.1 M PBS (pH = 7) solution at a scan rate of 100 mVs^−1^. (**b**) Linear regression plot of current vs. 4-NP concentration. (Accumulation time: 2 min, catalyst loading volume: 10 µL).

Linear Sweep Voltammetry (LSV) analysis is a simple and sensitive technique to sense the 4-NP over the Ag-ZnO/GCE-modified electrode. It is a simple and time saving technique due to its controllable sweep direction. Hence, we used the LSV technique for electrochemical sensing of 4-nitrophenol. The LSV responses were observed at different concentrations of 4-NP (10 µM to 500 µM) in 0.1 M PBS solution at a scan rate of 100 mVs^−1^. Figure 10 shows that the 4-NP oxidation peak current increased linearly by increasing the concentration of nitrophenol. In addition, the calibration was plotted with peak current against 4-NP concentration and is shown in Figure 10b. The Ag-ZnO modified electrode showed an excellent linear regression equation (R^2^ = 0.97206).

**Scheme 1:**
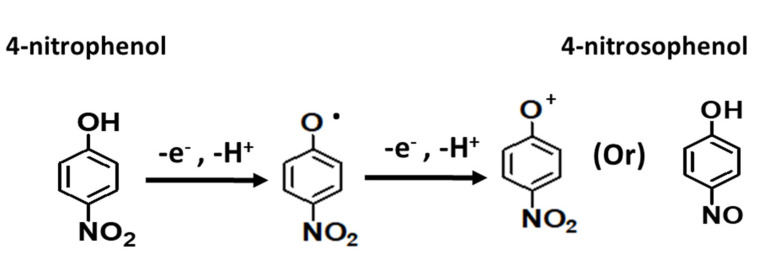
4-NP oxidation mechanism on the Ag-ZnO/GCE electrode.

Furthermore, the low detection limit, 3 µM (S/N = 3), was derived for Ag-ZnO/GCE electrode which revealed that the Ag-ZnO/GCE electrode could be the promising sensing electrode for 4-NP sensing. In addition, the oxidation mechanism was studied and is shown in Scheme 1. Generally, 4-NP oxidation followed an irreversible reaction at solution pH 7, which is a neutral medium. The 4-NP oxidation mechanism followed through phenolate radical formation and the results suggested that 4-nitrophenol oxidized to 4-nitrosophenol through phenolate radical formation which confirms the formation of intense oxidation peak at 0.9 V.

**Figure 11:**
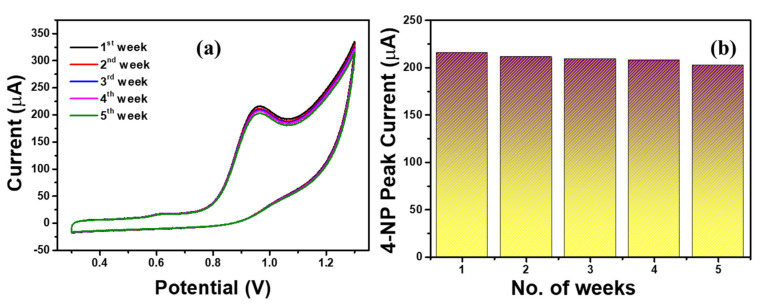
(**a**) Stability responses of Ag-ZnO/GCE (**b**) Ag-ZnO/GCE electrode response current in 500 µM 4-NP containing 0.1 M PBS (pH = 7) solution. (Accumulation time: 2 min, catalyst loading volume: 10 µL).

Generally, repeatability and stability of the sensor electrode was the key point in the electrochemical sensing application. Thus we analyzed the sensing stability of the Ag-ZnO/GCE electrode with 500 µM 4-NP containing 0.1 M PBS (pH = 7) solution at a scan rate of 100 mVs^−1^ for more than 5 weeks and the obtained results are shown in Figure 11. The Ag-ZnO/GCE electrode showed good sensing stability towards 4-NP over 5 weeks. From Figure 11b it was observed that, the peak current slightly decreased over three weeks. Hence, Ag-ZnO/GCE electrode could be the promising sensing electrode for the detection of 4-NP in real applications.

**Figure 12:**
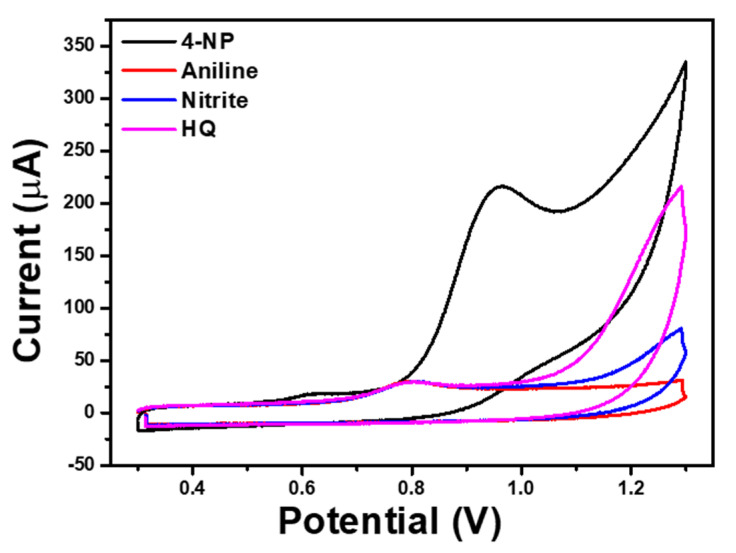
CV responses of Ag-ZnO/GCE in 500 µM 4-NP containing with 500 µM aniline, nitrite, hydroquinone in 0.1 M PBS (pH = 7) solution. (Accumulation time: 2 min, catalyst loading volume: 10 µL).

In addition, the interference study was analyzed to understand the selectivity of the Ag-ZnO/GCE electrode with 4-NP containing inorganic/organic interference chemicals as shown in Figure 12. From Figure 12, it can be seen that the noticeable change in the 4-NP oxidation peak current, which confirmed the poor selectivity of the Ag-ZnO/GCE electrode towards 4-NP with interference chemicals; however, the Ag-ZnO/GCE electrode showed high sensitivity towards 4-NP in the neutral medium. In addition, the sensitivity of Ag-ZnO/GCE electrode was compared to previously reported sensor electrodes in Table 1 [40,41,42,43].

## 3. Experimental Details

### 3.1. Materials

All chemicals such as silver nitrate, zinc nitratehexahydrate, hexamethylenetetramine, ammonium hydroxide, PBS, potassium ferrric cyanide and 4-nitrophenol were procured from Sigma-Aldrich and used as procured without further purification.

### 3.2. Synthesis of Ag-Doped Zno Nanoflowers Decorated with Nanosheets

Ag-doped ZnO nanoflowers were synthesized by the facile hydrothermal method. Typically, 0.1 M Zn(NO_3_)_2_·6H_2_O (40 mL) and 0.05 M AgNO_3_ (40 mL) solutions were mixed and vigorously stirred for 30 min, then an aqueous solution of 0.1 M HMTA (20 mL) was added and stirred for 30 min. A few drops of NH_4_OH were added to the resultant solution to maintain the pH = 11, then stirred again for 30 min. Consequently, the obtained solution was then poured into a Teflon-lined stainless steel autoclave and maintained at 160 °C for 6 h. After that, the autoclave was lowered to room-temperature, and finally whitish-yellow colored precipitate was filtered and washed with DI water and ethanol thoroughly, then dried at 60 °C for 3 h.

### 3.3. Characterizations of Ag-Doped ZnO Nanoflowers Decorated with Nanosheets

The synthesized Ag-doped ZnO nanoflowers decorated with nanosheets were examined using several techniques to understand the morphological, crystal, structural, compositional and surface properties. X-ray diffraction (PAN analytical Xpert Pro.) was employed to scrutinize the crystal properties, such as crystallinity, purity, phases, etc. The morphology, elemental composition and elemental mapping of the prepared Ag-doped ZnO nanoflowers decorated with nanosheets were obtained from field emission scanning electron microscopy (FESEM: JEOL-JSM-7600F) and transmission electron microscopy (TEM: JEOL-JEM-2100F) equipped with high-resolution TEM (HRTEM). To study the chemical compositions, the prepared nanoflowers were examined by Fourier transform infrared spectroscopy (FTIR: Spectrum-100), measured in the range of 450–4000 cm^−1^ at room-temperature using the KBr dispersion and palletization process. The optical properties were investigated by UV-Visible spectroscopy (UV Vis.: Perkin Elmer-UV-VIS-Lambda 950) in the range of 250–800 nm at room-temperature. X-ray photoelectron spectroscopy (XPS; AXIS-NOVA CJ109, Kratos Inc., San Diego, CA, USA) was utilized to analyze the binding energies and the existence of various oxidation states in the Ag-doped ZnO nanoflowers decorated with nanosheets.

### 3.4. Electrochemical Sensing Applications of Ag-Doped ZnO Nanoflowers Decorated with Nanosheets

All electrochemical studies such as cyclic voltammetry (CV), Linear sweep voltammetry (LSV) and Impedance analysis (EIS) were analyzed by using Princeton-applied research potential Stat 3 (AMETEK, Berwyn, PA, USA). For 4-NP sensor, three electrode systems with Ag-ZnO modified GCE, Pt and Ag/AgCl electrodes were used as working, counter and reference electrode respectively. 4-NP detection was analyzed in 0.1 M PBS (pH = 7) solution form the potential range of 0.25 to 1.3 V at a scan rate of 100 mVs^−1^. In addition, electrochemical activity and conductivity of Ag-ZnO/GCE electrode was analyzed in 5 mM ferri-ferro solution.

#### Fabrication of a Ag-Doped ZnO Nanoflowers-Modified Electrode

Ag-doped ZnO nanoflowers were modified by the drop casting method. Prior to drop casting, the GCE electrode was smoothly cleaned with 0.05 µm alumina paste on a polishing cloth to obtain a shiny surface. Then the electrode was washed with ethanol and water thoroughly. The 10 mg Ag-doped ZnO nanoflowers were dispersed with 1 mL of DI water through ultra-sonication. After that, 10 µL dispersion was drop coated on the precleared GCE electrode and allowed to evaporate the water molecule at room temperature for 2 h. Thus, Ag-ZnO/GCE was fabricated and stored at room temperature after the sensing experiment.

## 4. Conclusions

In summary, Ag-doped ZnO nanoflowers decorated with nanosheets were synthesized by facile hydrothermal process and characterized in detail using different characterization techniques. The detailed characterizations confirmed that the synthesized material was flower-shaped morphologies decorated with nanosheets and mainly exhibiting the wurtzite hexagonal phase of ZnO. The synthesized ZnO nanoflowers were used as a functional material to fabricate a highly sensitive and selective eco-toxic 4-nitrophenol chemical sensor over glassy carbon electrode. The fabricated chemical sensor exhibited more high-sensitivity and selectivity than the bare GCE electrode. An Ag-doped ZnO/GCE-based 4-nitrophenol chemical sensor exhibited a wide range of sensitivity from 10 µM to 500 µM with a good detection limit of 3 µM (S/N = 3). The observed results confirmed that the fabricated Ag-ZnO/GCE electrode could be the promising sensing electrode for various toxic chemicals.

## Figures and Tables

**Table 1:** Sensitivity comparison of the Ag-ZnO/GCE electrode on 4-NP.

Electrode	Method	Detection Potential (V)	Linear Range (µM)	LOD (µM)	Ref.
AuNP/RGO	DPV	−0.6	0.05–2.0	0.01	40
Ag NWs-PANI	DPV	−0.77	0.6–32	0.052	41
GCE/Fe_3_O_4_-Pt	DPV	−0.73	0.1–1.5	0.048	42
Ag/RGO	SWV	−0.5	10–101	0.0012	43
ɤ-Fe_2_O_3_-N-rGO hydrogel	DPV	−0.3, −0.6	1–1000	0.1	7
Ag-ZnO/GCE	LSV	0.96	10–500	3	This work

## Data Availability

Data is contained within the article.

## Sample Availability

The samples are available from the authors.
